# The Role of Inflammation in β-cell Dedifferentiation

**DOI:** 10.1038/s41598-017-06731-w

**Published:** 2017-07-24

**Authors:** Thierry M. Nordmann, Erez Dror, Friederike Schulze, Shuyang Traub, Ekaterine Berishvili, Charlotte Barbieux, Marianne Böni-Schnetzler, Marc Y. Donath

**Affiliations:** 1 Clinic of Endocrinology, Diabetes and Metabolism University Hospital Basel and Department Biomedicine, University of Basel, 4031 Basel, Switzerland; 2 Department of Surgery Cell Isolation and Transplantation Center, Geneva University Hospitals and University of Geneva, Geneva, Switzerland

## Abstract

Chronic inflammation impairs insulin secretion and sensitivity. β-cell dedifferentiation has recently been proposed as a mechanism underlying β-cell failure in T2D. Yet the effect of inflammation on β-cell identity in T2D has not been studied. Therefore, we investigated whether pro-inflammatory cytokines induce β-cell dedifferentiation and whether anti-inflammatory treatments improve insulin secretion via β-cell redifferentiation. We observed that IL-1β, IL-6 and TNFα promote β-cell dedifferentiation in cultured human and mouse islets, with IL-1β being the most potent one of them. In particular, β-cell identity maintaining transcription factor *Foxo1* was downregulated upon IL-1β exposure. *In vivo*, anti-IL-1β, anti-TNFα or NF-kB inhibiting sodium salicylate treatment improved insulin secretion of isolated islets. However, only TNFα antagonism partially prevented the loss of β-cell identity gene expression. Finally, the combination of IL-1β and TNFα antagonism improved insulin secretion of *ex vivo* isolated islets in a synergistic manner. Thus, while inflammation triggered β-cell dedifferentiation and dysfunction *in vitro*, this mechanism seems to be only partly responsible for the observed *in vivo* improvements in insulin secretion.

## Introduction

Decreased functional β-cell mass in the face of insulin resistance is a hallmark of type 2 diabetes (T2D)^1^. The proposed pathogenic mechanisms of β-cell dysfunction include glucotoxicity, lipotoxicity, endoplasmic reticulum stress, activation of the renin-angiotensin system and islet-associated β-amyloid^2–6^. Interestingly, all these pathogenic mechanisms elicit an inflammatory response involving the IL-1/nuclear factor kappa-light-chain-enhancer of activated B cells (NF-κB) pathway. Accordingly, treatment of patients with IL-1 antagonists or NF-κB inhibiting salsalate improves glycaemia in T2D^7, 8^. Other anti-inflammatory approaches, mainly TNFα antagonism, may equally improve glycaemia, although clinical development is less advanced despite convincing preclinical data^9, 10^.

Recently, Talchai and colleagues introduced the concept of β-cell dedifferentiation as an alternative mechanism of β-cell failure in T2D^11^. Thereby β-cells revert to progenitor-like cells expressing Neurogenin3 (*Ngn3*), POU domain class 5 transcription factor 1 (*Pou5f1*), *Nanog*, and *L-Myc*, along with reduced expression of key mature β-cell genes, such as Insulin2 (*Ins2*), Glucose transporter 2 (*Slc2a2*), pancreatic and duodenal homeobox 1 (*Pdx1*) and NK6 homeobox 1 (*Nkx6-1*)^12^. This loss of identity appears to be critically regulated by forkhead box O1 (*Foxo1*) and eventually leads to the inability of the β-cell to adequately produce and secrete insulin^11, 13^. In contrast to apoptosis, β-cell identity loss is a potentially reversible mechanism, implying that by means of redifferentation, β-cell function and glucose homeostasis could potentially be restored^14^. To date, the mechanisms causing β-cell identity loss are not well understood.

We hypothesized, that cytokine induced β-cell stress may trigger β-cell dedifferentiation in T2D. Indeed, cytokine stress causes dysfunctional β-cells characterized by reduced expression of β-cell genes and by impaired glucose-induced insulin secretion^15, 16^. However, most of these studies were conducted in the context of type 1 diabetes and with a mixture of multiple cytokines (e.g. IL-1β + IFN-γ^15^; IFN-γ ± IL-1β or TNFα^16^). Individual cytokine driven β-cell identity loss and potential redifferentation through anti-inflammatory treatment in type 2 diabetes models have not yet been assessed. We further hypothesized, that anti-inflammatory regimens might improve insulin secretion via β-cell redifferentiation or prevention of β-cell dedifferentiation.

We report, that IL-1β causes β-cell dedifferentiation *in vitro* and dampens insulin secretion capacity more prominently than TNFα and IL-6. While anti-IL-1β, anti-TNFα and NF-κB inhibiting sodium salicylate treatment improved glycaemia and augmented β-cell insulin secretion, only TNFα antagonism partially prevented the loss of β-cell identity gene expression. In addition, the combination of anti-IL-1β and anti TNFα *in vivo* improved insulin secretion of isolated islets.

## Results

### Immune cell infiltration in pancreatic islets of patients with type 2 diabetes

**Figure 1:**
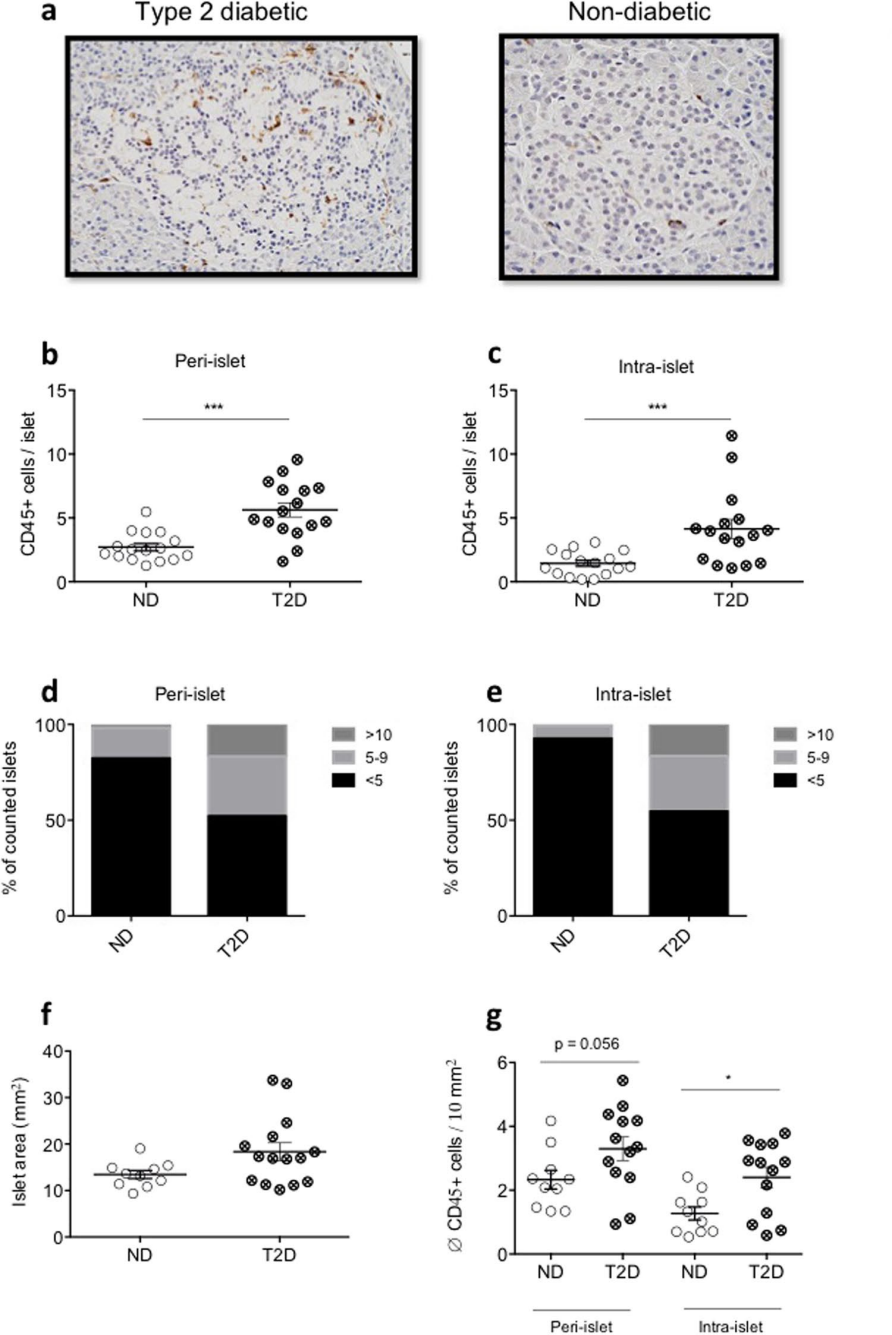
Pancreatic islets of patients with type 2 diabetes contain more CD45 + immune cells than islets of non-diabetic subjects. (**a**) Representative islet histology of type 2 diabetic (T2D) and non-diabetic (ND) subjects. Peri-islet and intra-islet CD45 + immune cell count (**b** and **c**, respectively) and distribution (**d** and **e**, respectively) in human pancreatic tissue sections of T2D and ND control subjects. Islet area (**f**) and islet-area-corrected average CD45 + immune cell count (**g**) in T2D and ND control subjects. (**b**–**e**) n = 17, 16 for T2D and ND subjects, respectively, (**f**,**g**) n = 14, 10 for T2D and ND subjects. n = 30–35 islets/subject were counted. ***P < 0.001. Statistical significance (P) was determined using the two-tailed Mann-Whitney U test. All error bars denote s.e.m.

Previous studies have shown the presence of macrophages in islets of patients with T2D^17, 18^. In order to determine the total number of infiltrating immune cells in islets of human type 2 diabetic subjects, paraffin embedded pancreatic tissue sections of 17 type 2 diabetic (T2D) and 16 non-diabetic (ND) subjects were assessed using the pan immune cell marker CD45. Both cohorts were matched for age and gender. Patients with T2D had a higher BMI and HbA1c levels, and numerically lower C-peptide levels compared to ND subjects (Table 1). Mean diabetes duration was 9.8 ± 3.1 years (Table 1). There were significantly more CD45^+^ immune cells around (T2D: 5.68 ± 0.52, ND: 2.72 ± 0.28 cells per islet; p = < 0.0001; Fig. 1a,b) and within (T2D: 4.10 ± 0.70, ND: 1.44 ± 0.24 cells per islet; p = 0.0003; Fig. 1a,c) pancreatic islets of T2D subjects. Interestingly, while most islets of T2D and ND subjects harbored less than five immune cells, type 2 diabetic islets contained significantly more islets with five to ten and more immune cells than the ND controls (Fig. 1d,e). Islet-area in patients with T2D tended to be increased compared to non-diabetic control individuals (p = 0.079; Fig. 1f). When corrected for islet-area, the average amount of immune cells within the islets were significantly elevated in type 2 diabetic subjects (p = 0.017), while the average amount of immune cells around the islets reached a p-value of 0.056 (Fig. 1g).

**Table 1:** Cohort characteristics of type 2 diabetic and non-diabetic patients analyzed in Fig. 1.

	T2D (n = 17)	ND (n = 16)	p-value
Age (y)	44.51 ± 3.682	43.52 ± 3.898	0.8799
BMI (kg/m^2^)	33.08 ± 1.674	27.44 ± 1.024	0.0201
HbA1c (%)	7.683 ± 0.4199	5.6 ± 0.1291	0.0043
C-peptide (ng/ml)	3.881 ± 1.541	7.341 ± 1.896	0.0988
Diabetes Duration (y)	9.75 ± 3.118		
Gender (m/f)	7/10	6/10

### Cytokines induce β-cell dedifferentiation

**Figure 2:**
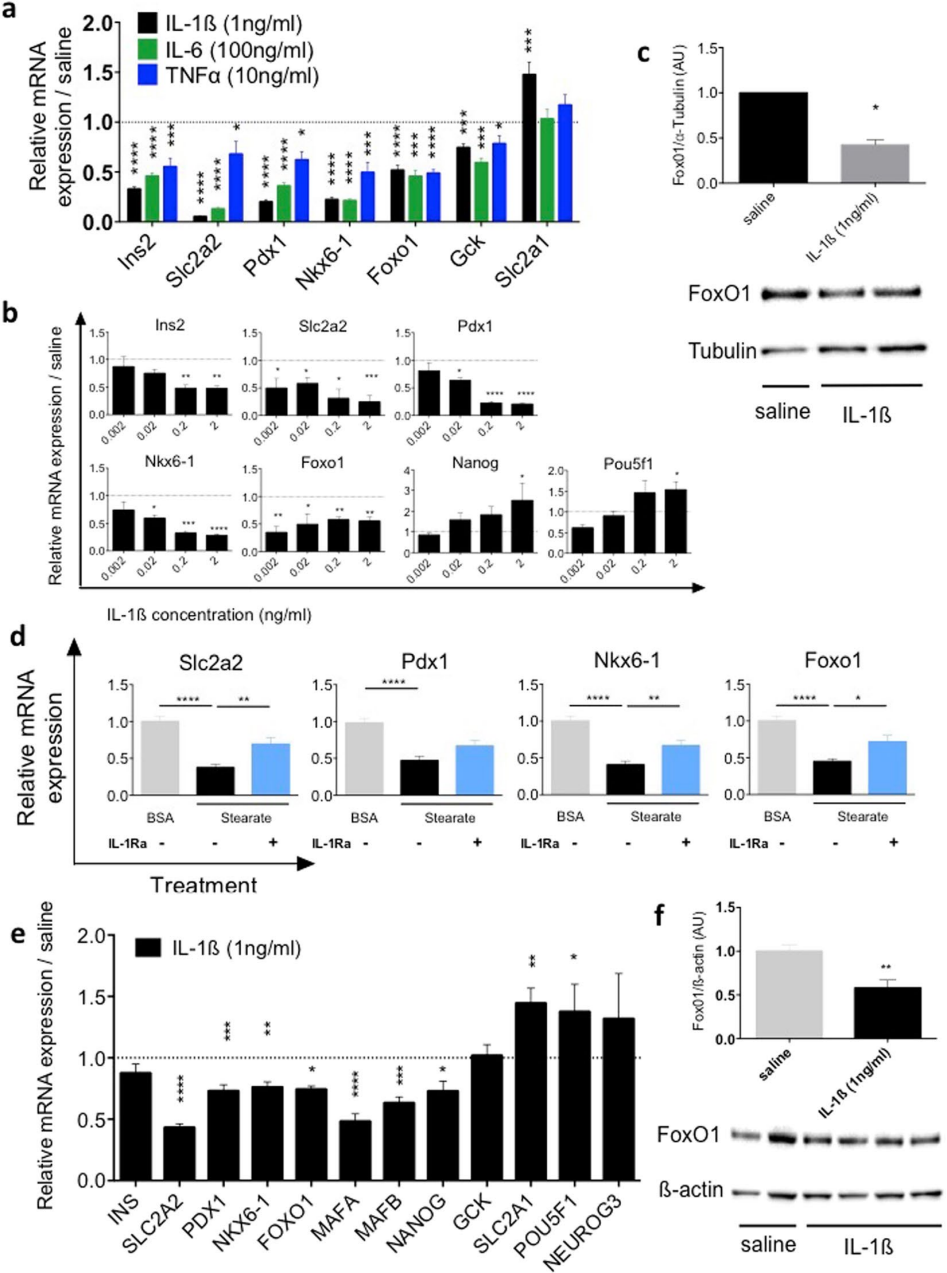
Cytokine induced β-cell dedifferentiation. (**a**) mRNA expression levels of Insulin (*Ins2*), GLUT2 (*Slc2a2*), *Pdx1* and *Nkx6-1*, *Foxo1*, glucokinase (*Gck*) and GLUT1 (*Slc2a1*) in mouse islets after 24 hours of treatment with 1ng/ml of IL-1β, 100ng/ml of IL-6 and 10 ng/ml of TNFα relative to solvent (dashed line). (**b**) mRNA expression levels in mouse islets after 24 hours of exposure to various concentrations of IL-1β relative to solvent (dashed line). (**c**) Protein quantification and a representative Western blot of FoxO1 in mouse islets after 24 hours treatment with IL-1β (1 ng/ml) or saline. (**d**) mRNA expression levels in mouse islets after 24 hours treatment with 0.25 mM of stearate in the absence (black bars) or presence of the IL-1 receptor antagonist IL-1Ra (1 μg/ml; blue bars) compared to solvent (BSA). (**e**) mRNA expression levels in human islets after 24 hours of IL-1β (1 ng/ml) treatment relative to solvent (dashed line). (**f**) Protein quantification and a representative Western blot of FoxO1 in human islets after 24 hours of IL-1β (1 ng/ml). (**a**) n = 12. (**b**) n = 9. (**c**) n = 3–5. (**d**) n = 14–16. (**e**) n = 9–10. (**f**) n = 6–8. All sample sizes represent the sum of 3 independent experiments. *P < 0.05, **P < 0.01, ***P < 0.001, ****P < 0.0001 of treatment group vs. untreated control. Statistical significance (P) was determined using the two-tailed Mann-Whitney U (Fig. a,c–f) and one-way ANOVA (Fig. b). All error bars denote s.e.m.

We next determined the effect of individual cytokines on β-cell identity gene expression of isolated pancreatic islets. Exposure of mouse islets to IL-1β, IL-6 or TNFα for 24 h decreased mRNA expression of *Ins2*, *Slc2a2*, β-cell transcription factors *Pdx1* and *Nkx6-1*, β-cell identity maintaining transcription factor *Foxo1* ^11^ and glucokinase (*Gck*), while GLUT1 (*Slc2a1*) was increased by IL-1β (Fig. 2a). Among the three cytokines tested, IL-1β had the most potent effect. Therefore, we treated mouse islets with increasing concentrations of IL-1β, causing a dose-dependent down-regulation of *Ins2*, *Slc2a2*, *Pdx1*, and *Nkx6-1* gene expression (Fig. 2b). Interestingly, *Foxo1* expression was already maximally suppressed at 0.002 ng/ml of IL-1β (Fig. 2b). In contrast, the β-cell progenitor transcription factors *Nanog* and *Pou5f1* were augmented with increasing IL-1β concentrations (Fig. 2b). Cycle threshold values of housekeeping genes *Gapdh*, *beta-actin* and *Rn18s* did not increase upon treatment with higher cytokine concentrations (Supplementary Fig. 2). Since FoxO1 has been implicated in the regulation of β-cell dedifferentiation^11^, we confirmed the decreased *Foxo1* expression by IL-1β also at protein level by Western Blotting (Fig. 2c). Next we tested whether free fatty acids (FFA) also cause β-cell dedifferentiation and whether it is IL-1 mediated. Treatment of islets with stearate, an abundant saturated free fatty acid in rodent circulation^19^, decreased *Slc2a2*, *Pdx1*, *Nkx6-1* and *Foxo1* expression. This effect was partially prevented by the addition of the IL-1Receptor antagonist IL-1Ra (Fig. 2d) while IL-1Ra alone had no effect compared to BSA (not shown). Finally, we examined if IL-1β suppresses β-cell identity markers in human pancreatic islets. Indeed, exposure of human islets to IL-1β for 24 h decreased mRNA expression of *Insulin*, *SLC2A2*, *PDX1*, *NKX6*-1, *FOXO1*, and β-cell transcription factors *MAFA*, *MAFB*, *NANOG*, while *GCK* remained unaffected, and *SLC2A1* and *POU5F1* were increased (Fig. 2e). As with mouse islets FOXO1 was also decreased at protein level in IL-1β treated human islets (Fig. 2f). Overall, these data show that the proinflammatory cytokines IL-1β, IL-6 and TNFα decrease β-cell differentiation markers with IL-1β having the strongest effects in mouse islets.

### *In vivo* anti-inflammatory treatment ameliorates diabetic phenotype and improves β-cell insulin secretion capacity

To evaluate if inflammation plays a role in β-cell identity loss *in vivo*, the effect of anti-IL-β, anti-TNFα and NF-κB-inhibiting sodium salicylate treatment on glycaemia and β-cell function was analyzed in the DIO/STZ-, DIO- and db/db-mouse models.

**Figure 3:**
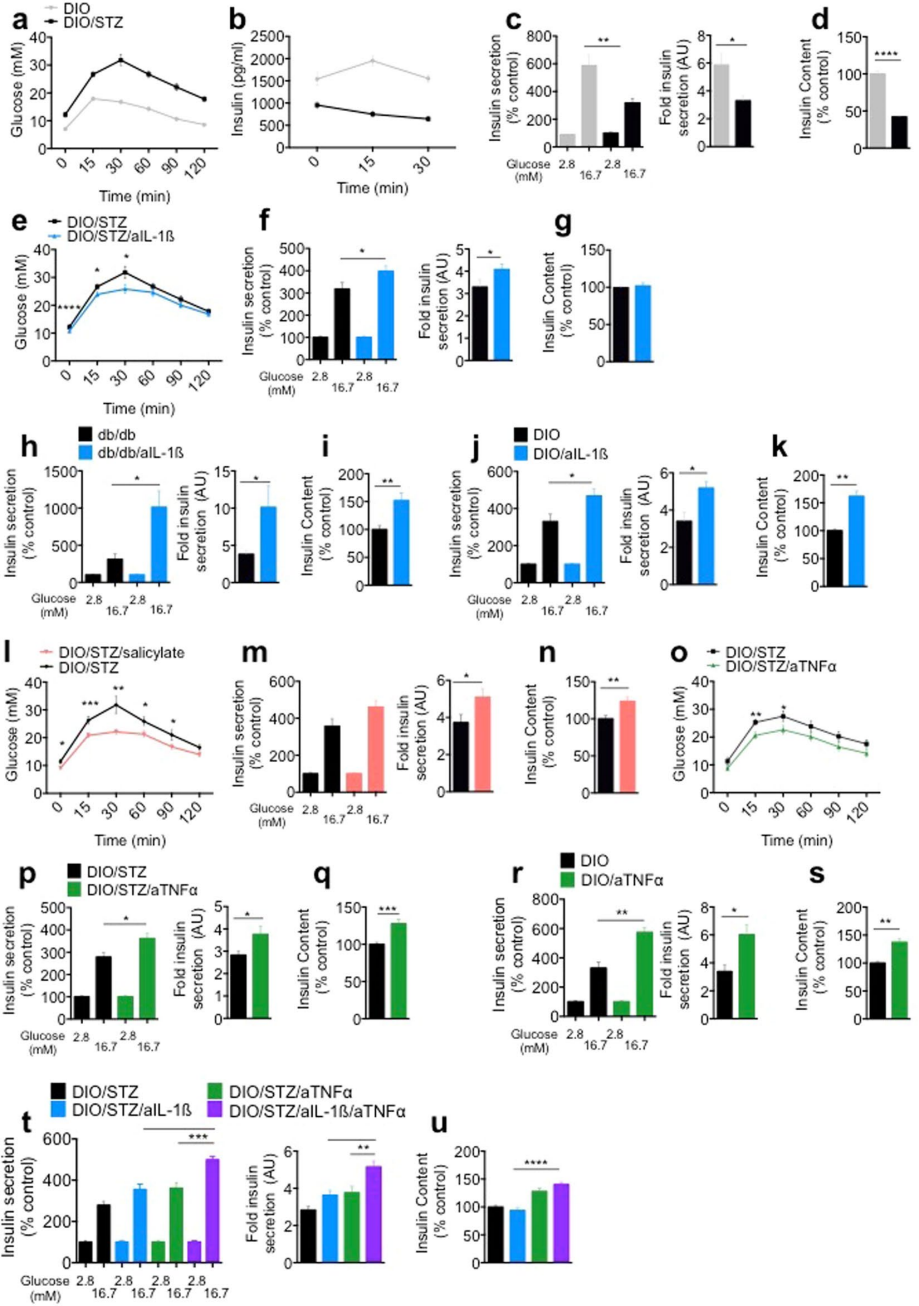
*In vivo* anti-inflammatory treatment ameliorates diabetic phenotype and improves β-cell insulin secretion capacity. (**a**) Glucose levels and (**b**) corresponding insulin levels following an ipGTT in DIO and DIO/STZ mice. (**c**,**d**) GSIS, corresponding fold insulin secretion and insulin content of isolated islets from DIO and DIO/STZ mice. (**e**) Glucose levels following an ipGTT in DIO/STZ mice ± anti-IL-1β (aIL-1β). (**f**,**g**) GSIS, corresponding fold insulin secretion and insulin content of isolated islets from DIO/STZ mice ± aIL-1β. (**h**–**k**) GSIS, corresponding fold insulin secretion and insulin content of isolated islets from (**h**,**i**) db/db mice ± aIL-1β treatment for 2 weeks and (**j**,**k**) DIO mice ± aIL-1β treatment for 8 weeks. (**l**) Glucose levels following an ipGTT in DIO/STZ mice ± sodium salicylate. (**m**,**n**) GSIS, corresponding fold insulin secretion and insulin content of isolated islets from DIO/STZ mice ± sodium salicylate treatment. (**o**) Glucose levels following an ipGTT in DIO/STZ mice ± anti TNFα (aTNFα) treatment. (**p**,**q**) GSIS, corresponding fold insulin secretion and insulin content of isolated islets from DIO/STZ mice ± aTNFα treatment. (**r**,**s**) GSIS and corresponding fold insulin secretion of isolated islets from DIO mice ± aTNFα treatment for 8 weeks. (**t**,**u**) GSIS, corresponding fold insulin secretion and insulin content from DIO/STZ mice ± aTNFα/aIL-1β treatment. (**a**,**b**) n = 19 each, 3 experiments. (**c**,**d**) n = 18 each, 3 experiments. (**e**) n = 19 each, 3 experiments. (**f**,**g**) n = 18 each, 3 experiments. (**h**,**i**) n = 6 each, 1 experiment. (**j**,**k**) n = 5 each, 1 experiment. (**l**) n = 14 each, 2 experiments. (**m**,**n**) n = 12 each, 2 experiments. (**o**) n = 11 each, 2 experiments. (**p**,**q**) n = 12 each, 2 experiments. (**r**,**s**) n = 6 each, 1 experiment. (**t**,**u**) n = 12 each, 2 experiments. n represents the number of mice in *in vivo* experiments and biological replicates in experiments with isolated islets. *P < 0.05, **P < 0.01, ***P < 0.001, ****P < 0.0001. Statistical significance (P) was determined using the two-tailed Mann-Whitney U (Fig. a-s) and one-way ANOVA (Fig. t,u). All error bars denote s.e.m. DIO, diet-induced obese; DIO/STZ, diet-induced obese/streptozotocin; ipGTT, intraperitoneal glucose-tolerance test.

4-week-old C57BL6/N mice were fed a HFD and either received a single injection of streptozotocin (STZ; 130 mg/kg) or sodium citrate at 8 weeks of age. This model mimics human type 2 diabetes by combining insulin resistance and a reduction in β-cell mass^20, 21^. Indeed, 2 weeks after STZ injection, DIO/STZ mice developed fasting hyperglycaemia, profound glucose intolerance (Fig. 3a) and decreased plasma insulin levels (Fig. 3b) during ipGTT compared to non-STZ treated mice. Insulin tolerance testing (ITT) did not reveal a difference in insulin sensitivity when corrected for baseline glucose (Supplementary Fig. 3a). In *ex vivo* isolated islets from DIO/STZ mice, glucose stimulated insulin secretion (GSIS) was impaired, along with a reduced fold stimulation of insulin secretion and reduced insulin content (Fig. 3c,d). The role of IL-1β was assessed in DIO/STZ mice using a mouse monoclonal antibody blocking IL-1β. After 2 weeks of treatment, glucose tolerance was improved (Fig. 3e). Although there were no differences in plasma insulin levels in anti-IL-1β antibody treated groups compared to controls (data not shown), *ex vivo* isolated islets from mice with IL-1β antagonism had an improved insulin secretion capacity in response to glucose and comparable insulin content (Fig. 3f,g). Similarly, IL-1β antibody treatment improved glucose induced insulin secretion and insulin content in islets isolated from db/db mice (Fig. 3h,i) and from mice fed a HFD (Fig. 3j,k) strengthening the concept of IL-1β driven impairment of insulin secretion. ITT pointed to enhanced insulin sensitivity in IL-1β antibody treated mice, although this was not statistically significant (Supplementary Fig. 3b). Given the potential benefit of inhibiting TNFα^9^ and NF-κB^8^ in the treatment of type 2 diabetes, the effect of NF-κB inhibiting sodium salicylate and TNFα inhibiting etanercept was assessed in the DIO/STZ and DIO mouse model. Two weeks of treatment with sodium salicylate improved glycaemia in the DIO/STZ mice (Fig. 3l), while no change in insulin levels could be detected in treatment and control groups (data not shown). *Ex vivo* isolated islets from mice treated with sodium salicylate had improved insulin secretion capacity and higher insulin content (Fig. 3m,n). Similarly, TNF inhibitor etanercept improved glycaemia in an ipGTT in DIO/STZ mice (Fig. 3o), while insulin levels remained comparable in treatment and control groups (data not shown). *Ex vivo* isolated islets from mice treated with etanercept showed improved insulin secretion and higher insulin content (Fig. 3p,q). Analogously, etanercept treatment improved glucose induced insulin secretion and insulin content in islets isolated from DIO mice (Fig. 3r,s). Insulin sensitivity was increased in etanercept treated DIO/STZ mice, although this was not statistically significant (Supplementary Fig. 3c). Given the positive effects of single anti-inflammatory treatment on glycaemia and insulin secretion, we assessed whether combinatorial treatment would lead to a synergistic effect. Indeed, the combination of IL-1β- and TNFα-antagonism resulted in an additive effect on insulin secretion and insulin content (Fig. 3t,u). In summary, treatment of mouse models of diabetes with IL-1β- and TNF-antagonists, and with sodium salicylate improved insulin secretion and glycaemia to comparable levels, with an additive effect on insulin secretion with the combination of IL-1β- and TNFα-antagonists.

### Impact of *in vivo* anti-inflammatory treatment on β-cell dedifferentiation

**Figure 4:**
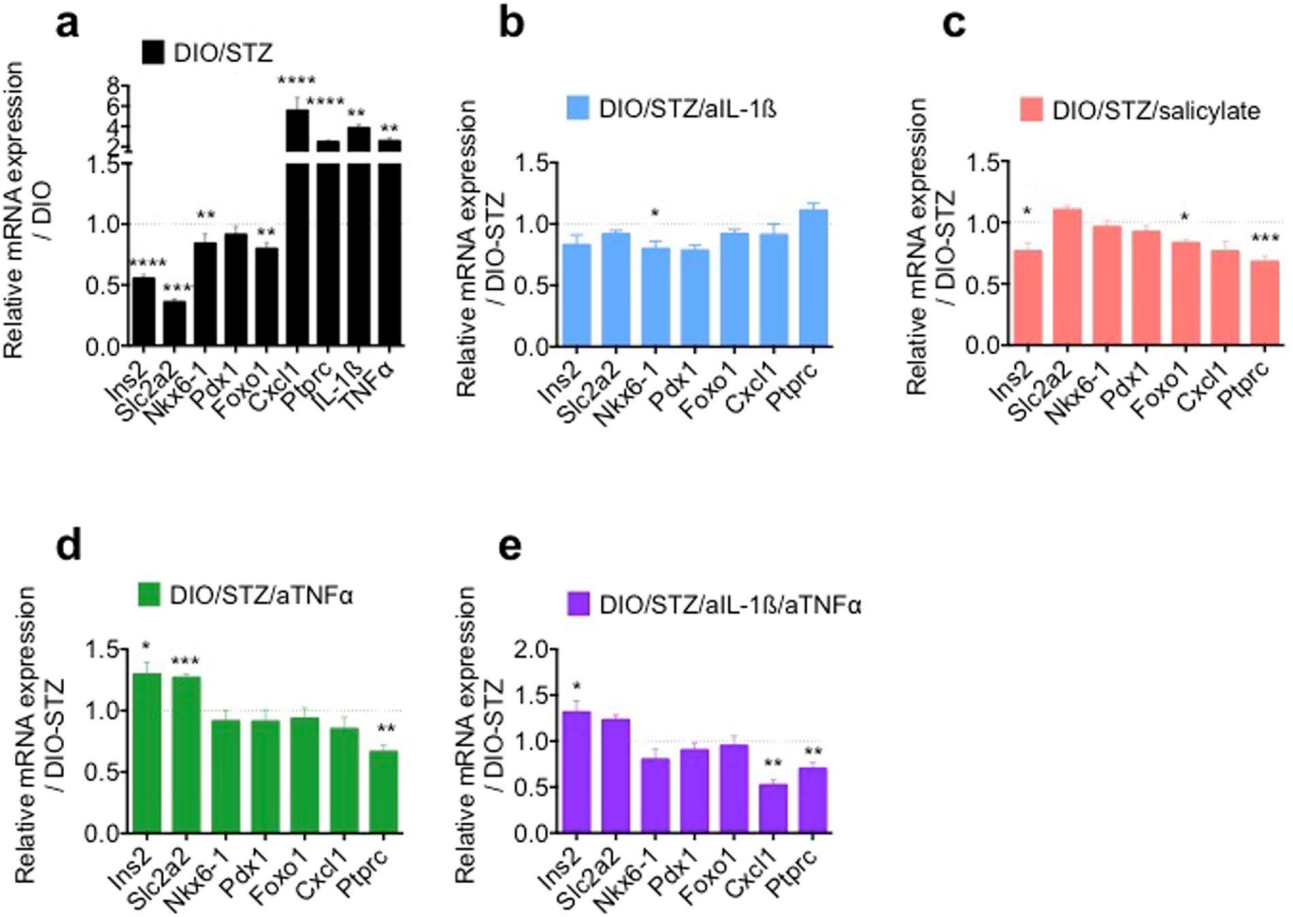
Impact of *in vivo* anti-inflammatory treatment on β-cell dedifferentiation. (**a**) Relative mRNA expression levels of isolated islets of DIO/STZ compared to DIO mice. (**b**) Relative mRNA expression levels of isolated islets from DIO/STZ mice ± aIL-1β treatment. (**c**) Relative mRNA expression levels of isolated islets from DIO/STZ mice ± salicylate treatment. (**d**) Relative mRNA expression levels of isolated islets from DIO/STZ mice ± aTNFα treatment. (**e**) Relative mRNA expression levels of isolated islets from DIO/STZ mice ± aTNFα/aIL-1β treatment. (**a**) n = 20 (DIO/STZ), 29 (DIO), 3 experiments, except for IL-1β and TNFα n = 5. (**b**) 18–20 each, 3 experiments. (**c**) n = 15–16 each, 2 experiments. (**d**) n = 7–10, 2 experiments. (**e**) n = 7–9, 2 experiments. *P < 0.05, **P < 0.01, ***P < 0.001, ****P < 0.0001. Statistical significance (P) was determined using the two-tailed Mann-Whitney U test. All error bars denote s.e.m. DIO, diet-induced obese; DIO/STZ, diet-induced obese/streptozotocin.

To assess the effect of IL-1β, TNFα or NF-κB inhibition on β-cell dedifferentiation we analyzed mRNA expression levels of isolated islets from the DIO/STZ animal studies. Islets isolated from DIO/STZ mice showed lower expression of *Ins2*, *Slc2a2*, *Nkx6-1* and *Foxo1* compared to HFD alone, while expression of inflammatory factors *Cxcl1*, *Ptprc*, *IL-1β* and *TNFα* were higher (Fig. 4a). Despite the clear contribution of IL-1β on dedifferentiation *in vitro*, IL-1β antagonism failed to prevent β-cell identity loss and the upregulation of inflammatory genes *in vivo* (Fig. 4b). Similarly, while sodium salicylate dampened the inflammatory gene response as reflected by down-regulation of the chemokine *Cxcl1* and of the leukocyte marker *Ptprc* (CD45), there was no increase of β-cell identity gene expression (Fig. 4c). Only TNFα-antagonism resulted in partial restoration β-cell identity gene expression and reduction of the inflammatory gene response within islets (Fig. 4d). Furthermore, the combination of IL-1β- and TNFα-antagonism significantly reduced the inflammatory gene response, while the expression of β-cell identity genes were similar to TNFα-antagonism alone (Fig. 4e).

## Discussion

In the present study we investigated the contribution of proinflammatory cytokines in β-cell dedifferentiation and its prevention by anti-inflammatory agents. We observed that mainly IL-1β, but also IL-6 and TNFα decreased the expression of differentiation markers in cultured islets. Treatment of diabetic animal models with anti-inflammatory agents improved insulin secretion in isolated islets. However, only TNFα-antagonism resulted in partial restoration of β-cell differentiation with no additive effects when combined with IL-1β antagonism, despite reduced inflammatory gene expression. Therefore, while inflammation may cause β-cell dedifferentiation, it remains unclear how relevant this mechanism is *in vivo*.

Emphasizing the importance of immune cell infiltration in type 2 diabetes we show that CD45^+^ immune cells accumulate within and around pancreatic islets of type 2 diabetic subjects, histologically confirming recent data acquired by flow cytometry^22^. This analysis represents the largest histological analysis in an unbiased T2D cohort to date, and confirms previous histological quantification that have been made in potentially biased populations, including specimens for tumor biopsies or pancreatitis, and which only have quantified CD68^+^ macrophages^17, 18^.

While IL-1β, IL-6 and TNFα, cytokines involved in type 2 diabetes^7, 9, 23^, provoked downregulation of key β-cell genes, such as *Ins2*, *Slc2a2*, and *Pdx1*, the cytokine IL-1β did so most profoundly. This effect was dose-dependent with β-cells upregulating pluripotency markers at higher IL-1β concentrations, further strengthening the concept of cytokine driven β-cell dedifferentiation. Importantly, we show that exposure of human pancreatic islets to IL-1β also caused broad β-cell dedifferentiation, characterized by both loss of essential β-cell genes as well as the upregulation of pluripotency marker *POU5F1*. Putting our data into a pathophysiological context, we demonstrated that FFA-induced β-cell identity loss was partially reversible by IL-1Ra.

IL-β induces apoptosis following a 2–4 day exposure to concentrations of 2 ng/ml and higher, while lower concentrations do not induce apoptosis^24, 25^. The number of apoptotic cells induced by IL-1β at 2 ng/ml is below 1%. In contrast, the observed decrease of β-cell identity genes, such as *Slc2a2* and *Foxo1* was greater than 50% already at concentrations of 0.002 ng/ml and after only 24 h. In addition, housekeeping genes (*Gapdh*, *beta-actin* and *Rn18s*) expression did not decrease upon treatment with higher cytokine concentrations. It is therefore unlikely that cell death is responsible for the marked suppression of β-cell identity genes.

The predominant role of IL-1β in β-cell dedifferentiation is compatible with the particularly high expression level of the IL-1 receptor in β-cells^2^. It is therefore surprising that IL-1 antagonism *in vivo* failed to impact β-cell differentiation while TNFα antagonism partially restored impaired β-cell identity gene expression. Interestingly both IL-1β and TNFα are potent drivers of the NF-kB pathway, inducing NO production^26^. To examine if activation of the NF-kB pathway may drive dedifferentiation *in vivo*, we used sodium-salicylate treatment, a potent IKK- β inhibitor^27^. However, while glycemic improvement was noted, no beneficial effect on the reduction of β-cell identity genes was observed, arguing against a major role of the NF-kB pathway in β-cell dedifferentiation *in vivo*. Possibly, indirect effects related to insulin resistance had a stronger impact on β-cell differentiation. Indeed, the role of TNFα on insulin resistance is well described^9, 10^ and we observed some improvement in insulin tolerance, although not reaching statistical significance. Furthermore, even the combinatorial anti-inflammatory treatment, targeting both IL-1β and TNFα, and resulting in an additive effect on the inflammatory gene response, was not superior to TNFα-antagonism alone with respect to restoration of β-cell identity loss.

In summary, we demonstrated that cytokines drive pancreatic islet β-cell identity loss *in vitro* in both human and mouse islets. Anti-inflammatory treatment improved glycemic burden *in vivo* and improved insulin secretion capacity *ex vivo* while only modestly affecting β-cell dedifferentiation. Thus, while our results contribute to the further understanding of β-cell dedifferentiation, a suitable and effective target preventing dedifferentiation or even promoting redifferentiation *in vivo*, remains to be found. Furthermore, our findings revealed that the beneficial role of anti-inflammatory treatment of type 2 diabetes is unlikely the consequence of β-cell redifferentiation.

## Research Design and Methods

### Animal Experiments

Male C57Bl6/N were obtained from Charles River or from in house breeding using Charles River C57BL6/N mice. For the diet-induced obese (DIO)/Streptozotocin (STZ) experiments, C57BL6/N mice were fed a high-fat diet (HFD; D12331; Research Diets) starting at 4 weeks of age and a single i.p. injection of STZ (130 mg/kg; Sigma Aldrich) or vehicle control was applied at 8 weeks of age. The following 3 anti-inflammatory treatments were initiated at 8 weeks of age for 2 weeks: 1) IL-1β antagonism was performed by intraperitoneal (i.p.) injection of murine anti–IL-1β antibody [same specificity as canakinumab^28^, provided by Novartis (Basel, Switzerland)] or saline control once weekly at a dose of 10 mg/kg as suggested by the manufacturer. 2) Sodium salicylate (S3007; Sigma-Aldrich) was incorporated into the high-fat diet at a dose of 4 g/kg by the manufacturer (Research Diets) as described in ref. 29. 3) Etanercept (Enbrel®, Amgen) or saline control was administered subcutaneously 3 times per week at a dose of 20 mg/kg. For the DIO mouse experiments, 8-week-old C57BL6/N mice were put on a HFD (D12331; Research Diets, New Brunswick, NJ) and anti-inflammatory treatment (murine anti–IL-1β antibody, etanercept or saline, as mentioned above) was initiated after 10 weeks of HFD for a total of 8 weeks before sacrifice. The homozygote leptin-receptor deficient db/db mice (BKS.Cg-Dock7^m^+/+Lepr^db^/J; #000642) on a C57BLKS/J background were obtained from Charles River at the age of 5 weeks. Anti-inflammatory treatment (murine anti–IL-1β antibody or saline, as mentioned above) was initiated at the age of 6 weeks for 2 weeks. All animal studies were approved by the cantonal authority of Basel (Kantonales Veterinäramt Basel-Stadt, Basel, Switzerland). All animal experiments were done according to the Swiss Animal welfare law, institutional guidelines and the cantonal authority of Basel (Kantonales Veterinäramt Basel-Stadt, Basel, Switzerland).

### Mouse islet isolation

Animals were euthanized using CO_2_. After clamping the bile duct in proximity of the liver, the pancreas was perfused through the pancreatic duct via the sphincter of Oddi with a collagenase solution (1.4 g/l collagenase IV [Worthington]), removed and digested in the same solution for 28 min at 37 °C. After incubation, the tissue was dissociated by shaking and islets were harvested by centrifugation and sequential filtration through 500 μm and 70 μm cell strainers followed by handpicking under a microscope. Islets were either lysed for mRNA extraction or cultured on extracellular matrix-coated petri dishes or 24-well plates (Novamed, Israel) in RPMI-1640 (GIBCO) medium containing 11.1 mM glucose, 100 units/ml penicillin, 100 μg/ml streptomycin, 2 mM glutamax, 50 μg/ml gentamycin, 10 μg/ml fungison and 10% FCS (Invitrogen) for 48 hours before starting subsequent experiments. Mouse recombinant IL-1β, IL-6 or TNFα (all from R&D Systems, biotechne) were used at different concentrations as noted within the experiment for 24 hours before further analysis. Stearate (S3381, Sigma-Aldrich) was conjugated with low-endotoxin BSA (A8806, Sigma-Aldrich) at a molar ratio of 6:1 and the concentration was measured at the central laboratory of the University Hospital of Zuerich, Switzerland. BSA conjugated free fatty acids or BSA only control was used at 0.25 mM for 24 hours before further analysis. IL-1Ra (Anakinra, Kineret, Amgen) at a concentration of 1 µg/ml was added 30 minutes prior to other cytokine treatment.

### Human pancreatic islets

Human islets were isolated from pancreata of cadaver organ donors in accordance with the Swiss Ethical Committee and were provided by the islets for research distribution program through the European Consortium for Islet Transplantation, under the supervision of the Juvenile Diabetes Research Foundation (31-2012-783). All methods were performed in accordance with the relevant guidelines and regulations. Islets were cultured in CMRL-1066 medium containing 5 mmol/l glucose, 100 units/ml penicillin, 100 μg/ml streptomycin, 2 mM glutamax and 10% FCS (Invitrogen) on extracellular matrix-coated petri dishes or 24-well plates (Novamed, Israel). Human recombinant IL-1β (R&D Systems, biotechne) was used at 1 ng/ml for 24 hours before further analysis.

### Glucose tolerance tests

All mice were fasted in the morning for 6 h. Thereafter, 2 g glucose per kg body weight was injected intraperitoneally (ipGTT). Before injection (time point 0 min), 15 and 30 min after glucose administration, 25 μl of blood was collected from the tail-vain into EDTA-containing eppendorf tubes on ice for later insulin determination. At all time points (0, 15, 30, 60, 90, 120 min), the average blood glucose level of two measurements was determined using a glucose-meter (Freelite; Abbott Diabetes Care Inc.).

### Insulin tolerance tests

All mice were fasted in the morning for 3 h. Thereafter, 1 U Insulin (Novorapid, Novo Nordisk) per kg body weight was injected intraperitoneally. Glucose measurements were performed at 0, 30, 60, 90 and 120 min after insulin injection, as described above.

### Hormone measurements

Insulin concentrations were measured by electro-chemiluminescence, using mouse/rat insulin kits (Mesoscale Discovery) according to the manufacturers instructions.

### Glucose-induced insulin secretion assay

For *in vitro* glucose-stimulated insulin secretion experiments, 20 islets/well were seeded in 24-well plates and cultured for 48 hours. Culture supernatants were collected to determine chronic insulin release. Islets were then pre-incubated for 30 minutes in modified Krebs-Ringer bicarbonate buffer (KRB; 115 mM NaCl, 4.7 mM KCl, 2.6 mM CaCl_2_ 2H_2_O, 1.2 mM KH_2_PO_4_, 1.2 mM MgSO_4_ 7H_2_O, 10 mM HEPES, 0.5% bovine serum albumin, pH 7.4) containing 2.8 mM glucose at 37 °C. KRB was then replaced by fresh KRB containing 2.8 mM glucose and collected after 1 hour to determine the basal insulin release. This was followed by 1-hour incubation in KRB containing 16.7 mM glucose to determine the stimulated insulin release. Finally, islet cells were lysed and insulin extracted with 0.18 N HCl in 70% ethanol overnight at 4 °C for determination of insulin content. The stimulatory index (fold insulin secretion) is the quotient of the stimulated and basal insulin release per hour.

### Immunohistochemical staining

Human paraffin-embedded pancreatic tissue slides were received from the Network for Pancreatic Organ Donors with Diabetes (nPOD), a collaborative type 1 diabetes research project sponsored by JDRF. Organ Procurement Organizations partnering with nPOD to provide research resources are listed at http://www.jdrfnpod.org/for-partners/npod-partners/. nPOD tissue samples are classified as “Non-Human Subjects” according to the University of Florida IRB. Approved by the Basel Ethical Committee, no Ethical Board review was required. Tissue slides were deparaffinized, rehydrated and stained with mouse anti-human CD45 (1:100, overnight at 4 °C; DAKO, M0701) and biotinylated anti-mouse immunoglobulins (1:200, 60 min at room temperature, DAKO, E0354) and visualized with DAB (2 min, DAKO, K3467). Pictures of 30–35 islets per section were obtained and CD45+ cells within and around islets were counted. Intra-vessel CD45+ cells were excluded. Staining, photo acquirement and cell counting was done in a blinded manner and unblinding occurred after counting all samples.

### RNA extraction and qPCR

RNA of isolated mouse and human islets was extracted with the NucleoSpin RNA II Kit (Machery Nagel, Germany). cDNA was prepared with random hexamers and Superscript II Reverse Transcriptase (Invitrogen). For quantitative PCR, the real time PCR system 7500 (Applied Biosystems) and the following TaqMan assays were used (*Slc2a2:* Mm00446230_g1, *SLC2A*2: Hs01096908_m1; *Ins2*: Mm00731595_g1; *Pdx1*: Mm00435565_m1, *PDX1*: Hs00236830_m1; *Foxo1*: Mm00490671_m1, *FOXO1*: Hs01054576_m1; *Cxcl1*: Mm04207460_m1, *CXCL1*: Hs01100741_m1; *Ptprc*: Mm01293577_m1; *Nkx6*-1: Mm00454961_m1; *Il1b*: Mm00434228_m1; *TNFα*: Mm00443258_m1; *Pou5f1*: Mm03053917_g1; *Nanog*: Mm02019550_s1; *Gck*: Mm00439129_m1, *GCK*: Hs01564555_m1; *Slc2a1*: Mm00439129_m1, *SLC2A2:* Hs00892681_m1; all from Applied Biosystems). Alternatively, SYBR Green Real time mastermixes (Promega) with the following primers (all from Microsynth) were used: Mm*Slc2a2*: (F) TCAGAAGACAAGATCACCGGA, (R) GCTGGTGTGACTGTAAGTGGG; Mm*Gck*: (F) GCTGGTGTGACTGTAAGTG-GG, (R) GCAACATCTTTACACTGGCCT; Mm*FoxO1*: (F) GTACG-CCGACCTCATCACCA, (R) TGCTGTCGCCCTTATCCTTG; Mm*Pdx1*: (F) CCCCAGTTTACAAGCTCGCT, (R) CTCGGTTCCATTCGGGAAAGG; Mm*Ins2* (F) TGGCTTCTTCTACACACCCAAG, (R) ACAATGCCACGCTTCTGCC; Mm*Nkx6-1*: (F) TCAGGTCAAGGTCTGGTTCC, (R) CGATTTGTGC-TTTTTCAGCA. Data were normalized with the housekeeping gene HPRT for human islet mRNA and with GAPDH for mouse islet mRNA and relative expression was quantified using the comparative 2^−ΔΔCT^ method.

### Western Blotting

Human or mouse islets were washed on ice with PBS, and frozen tissue was thawed on ice before protein was extracted using a lysis buffer (20 mM Tris pH 7.5, 1% Triton X-100, 150 mM NaCl, 10% glycerol, protease and phosphatase inhibitors) and subsequent centrifugation. Soluble protein concentration was measured with the BCA protein assay kit (Thermo Fisher Scientific) and adjusted to the desired concentration in a protein sample buffer (NuPage LDS Sample Buffer; Life Tech). After denaturation and addition of anti-reducing agent (Life Tech), 10–50 μg of protein per lane was loaded, separated by electrophoresis (NuPage 4.12% Bis-Tris Gel; Life Technologies) and wet-transferred onto nitrocellulose membranes. Successful transfer was verified using Ponceau staining. Membranes were blocked for 30 minutes on a shaker at room temperature, with TBS-0.1%Tween containing either 5% dry milk or 3% BSA, followed by overnight (anti-FoxO1, 1:1000, CST #2880, 4 °C) or 1 hour (anti-β actin, anti-Tubulin, 1:5000, Sigma, RT) incubation. Membranes were then washed three times prior to incubation with the corresponding secondary antibodies (1:10000, all from santa-cruz) for 1 hour, at room temperature. After three washing steps, the stained proteins were visualized using ECL Blotting Substrate for HRP (Bio Rad). Blots were analyzed using image lab 4.1 software (Bio-Rad) and normalized with the housekeeping protein Tubulin for mouse proteins and β actin for human proteins according to standard protocol.

### Statistical analysis

Statistical analysis was performed using GraphPad Prism 6.0 (Graphpad Software Inc., San Diego, CA). Data are presented as mean ± SEM and were analyzed using the two-tailed Mann-Whitney U tests or one-way ANOVA as specified. Differences were considered statistically significant when P < 0.05.

## Electronic supplementary material

Supplementary Dataset 1
