# Supplementary material for: The Role of Inflammation in β-cell Dedifferentiation
Source: Sci Rep. 2017 Jul 24;7:6285. doi: 10.1038/s41598-017-06731-w (PMC5524956; doi:10.1038/s41598-017-06731-w)
Supplement: Supplementary file 1 — Supplementary Dataset 1 [file 41598_2017_6731_MOESM1_ESM.pdf]

Supplementary Files -

The Role of Inflammation in β-cell Dedifferentiation

Thierry M. Nordmann[1,3], Erez Dror[1], Friederike Schulze[1], Shuyang Traub[1], Ekaterine Berishvili[2], Charlotte Barbieux[2], Marianne Böni-Schnetzler[1], Marc Y. Donath[1]

[1]Clinic of Endocrinology, Diabetes and Metabolism University Hospital Basel and Department Biomedicine, University of Basel, 4031 Basel, Switzerland

[2]Department of Surgery Cell Isolation and Transplantation Center, Geneva University Hospitals and University of Geneva, Geneva, Switzerland.

[3]Corresponding author: t.nordmann@unibas.ch, # 0041’61’265 25 25

Suppl. Fig. 2.

a

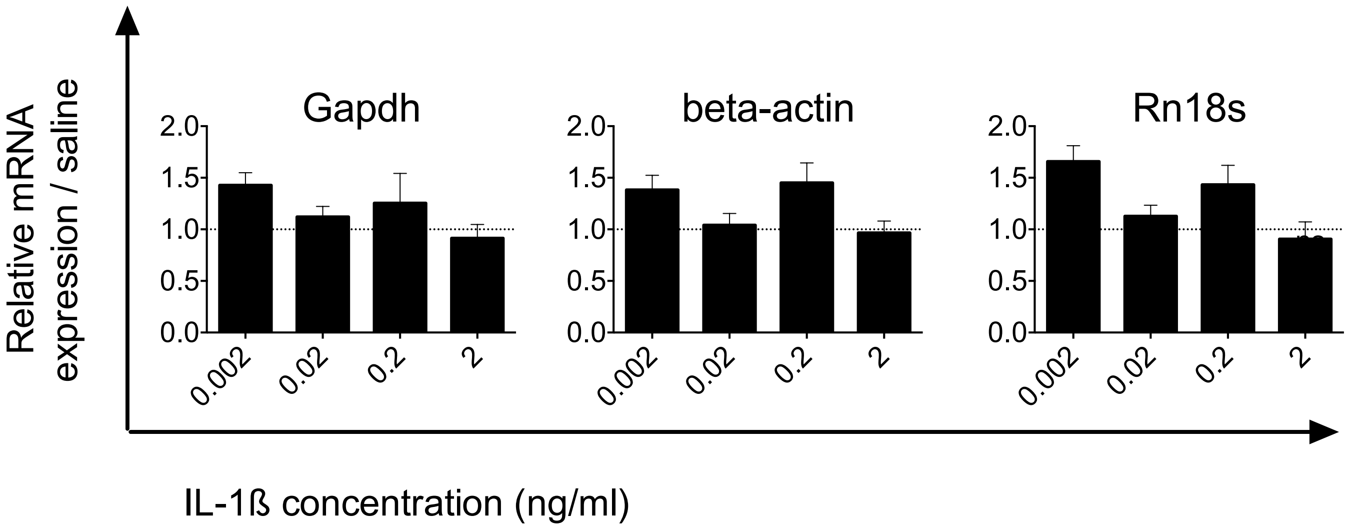

Gapdh
beta-actin
Rn18s
Relative mRNA expression / saline
2.0
1.5
1.0
0.5
0.0
0.002
0.02
0.2
2
IL-1ß concentration (ng/ml)

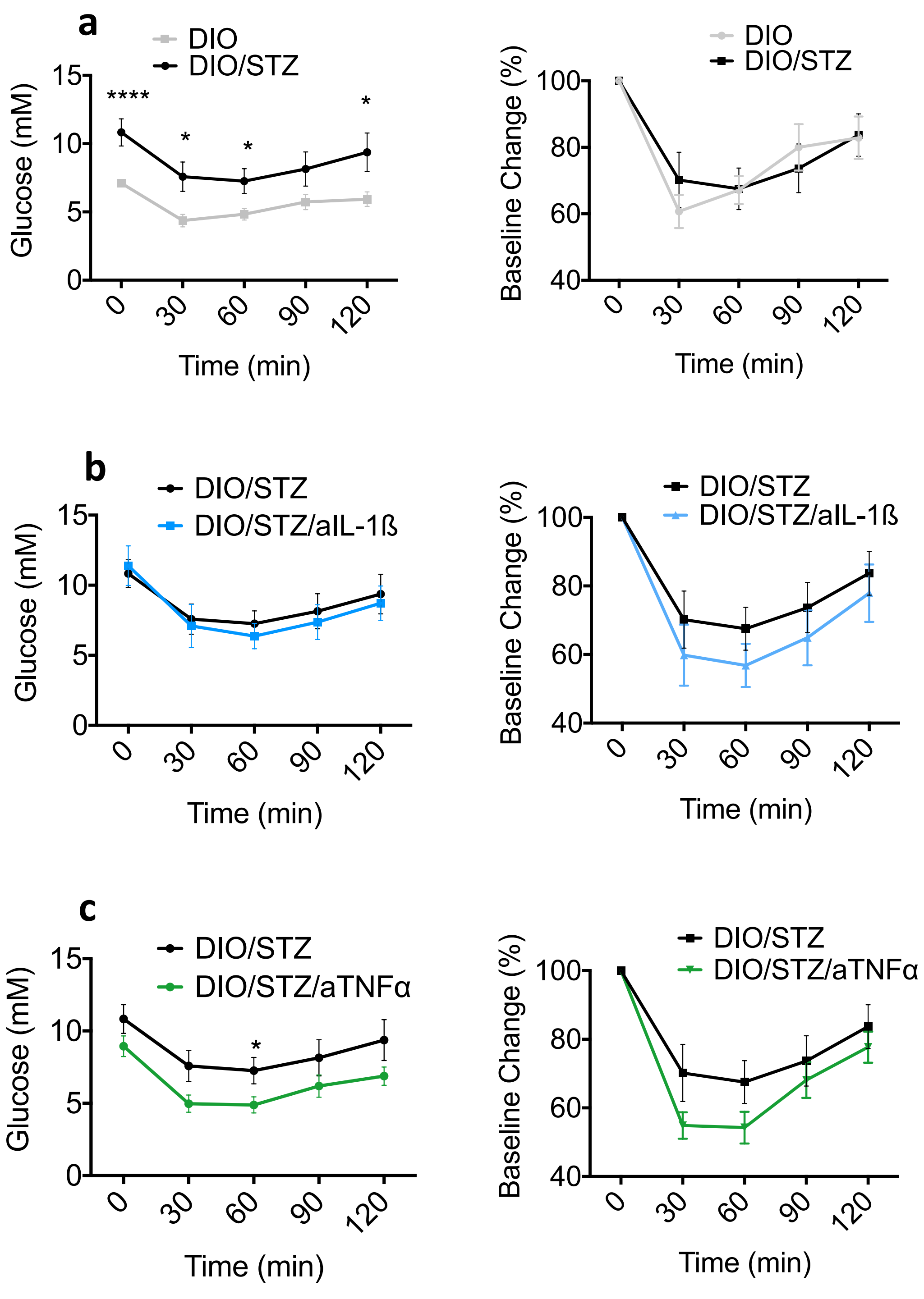
a
DIO
DIO/STZ
Glucose (mM)
****
*
*
*
Time (min)
Baseline Change (%)
b
DIO/STZ
DIO/STZ/aIL-1ß
c
DIO/STZ
DIO/STZ/aTNFα

Suppl. Fig. 3.

Supplementary Fig. 2.

Housekeeping gene expression upon various doses of IL-1 β

mRNA expression levels of the housekeeping genes *Gapdh*, *beta-actin* and *Rn18s* in mouse islets after 24 hours of exposure to various concentrations of IL-1 β relative to solvent (dashed line).

(a-b) n=9-10 per condition, representing the sum of 3 independent experiments. (c) n=6-7 per condition, representing the sum of 2 independent experiments. *P < 0.05, **P < 0.01, ***P < 0.001, ****P < 0.0001 of treatment group vs. untreated control. Statistical significance (P) was determined using the two-tailed Mann-Whitney U. All error bars denote s.e.m.

Supplementary Fig. 3.

Insulin tolerance test upon in vivo anti-inflammatory treatment

Glucose levels and corresponding change from baseline following an ITT in (a) DIO and DIO/STZ, (b) DIO/STZ ± aIL-1 β treatment and (c) DIO/STZ ± aTNF α treatment.

n=10 each, pooled from 2 independent experiments. n represents the number of mice. *P < 0.05, **P < 0.01, ***P < 0.001, ****P < 0.0001. Statistical significance (P) was determined using the two-tailed Mann-Whitney U. All error bars denote s.e.m. DIO, diet-induced obese; DIO/STZ, diet-induced obese / streptozotocin; ITT, insulin-tolerance test.
